# X-ray Computed Tomography Procedures to Quantitatively Characterize the Morphological Features of Triply Periodic Minimal Surface Structures

**DOI:** 10.3390/ma14113002

**Published:** 2021-06-01

**Authors:** Sergei Evsevleev, Tatiana Mishurova, Dmitriy Khrapov, Aleksandra Paveleva, Dietmar Meinel, Roman Surmenev, Maria Surmeneva, Andrey Koptyug, Giovanni Bruno

**Affiliations:** 1 Bundesanstalt für Materialforschung und -prüfung (BAM), Unter den Eichen 87, 12205 Berlin, Germany; tatiana.mishurova@bam.de (T.M.); dietmar.meinel@bam.de (D.M.); giovanni.bruno@bam.de (G.B.); 2 Physical Materials Science and Composite Materials Centre, Research School of Chemistry & Applied Biomedical Sciences, National Research Tomsk Polytechnic University, 30 Lenina Avenue, 634050 Tomsk, Russia; dah8@tpu.ru (D.K.); aleksandra-paveleva@mail.ru (A.P.); rsurmenev@mail.ru (R.S.); surmenevamaria@mail.ru (M.S.); andrey.koptyug@miun.se (A.K.); 3 Department of Engineering and Sustainable Development, Mid Sweden University, Akademigatan 1, SE-831 25 Östersund, Sweden; 4 Institute of Physics and Astronomy, University of Potsdam, Karl-Liebknecht-Str. 24-24, 14476 Potsdam, Germany

**Keywords:** metamaterials, functionally graded porous structure, triply periodic minimal surface structures, roughness analysis, powder removal, deep learning segmentation

## Abstract

Additively manufactured (AM) metallic sheet-based Triply Periodic Minimal Surface Structures (TPMSS) meet several requirements in both bio-medical and engineering fields: Tunable mechanical properties, low sensitivity to manufacturing defects, mechanical stability, and high energy absorption. However, they also present some challenges related to quality control, which can prevent their successful application. In fact, the optimization of the AM process is impossible without considering structural characteristics as manufacturing accuracy, internal defects, as well as surface topography and roughness. In this study, the quantitative non-destructive analysis of TPMSS manufactured from Ti-6Al-4V alloy by electron beam melting was performed by means of X-ray computed tomography (XCT). Several advanced image analysis workflows are presented to evaluate the effect of build orientation on wall thicknesses distribution, wall degradation, and surface roughness reduction due to the chemical etching of TPMSS. It is shown that the manufacturing accuracy differs for the structural elements printed parallel and orthogonal to the manufactured layers. Different strategies for chemical etching show different powder removal capabilities and both lead to the loss of material and hence the gradient of the wall thickness. This affects the mechanical performance under compression by reduction of the yield stress. The positive effect of the chemical etching is the reduction of the surface roughness, which can potentially improve the fatigue properties of the components. Finally, XCT was used to correlate the amount of retained powder with the pore size of the functionally graded TPMSS, which can further improve the manufacturing process.

## 1. Introduction

Additive manufacturing (AM) techniques, such as electron beam melting (EBM) or laser powder bed fusion (LPBF) allow producing geometrically complex structures, including near net-shape ones. Triply Periodic Minimal Surface Structures (TPMSS) are one of the topologies, which can be produced only by AM technologies. TPMSS have mean curvature equal to zero at every point and exhibit periodicity in three independent space directions [1].

One of the most promising applications of TPMSS is tissue engineering, since they possess mechanical characteristics (e.g., Young’s modulus and strength) that can be tuned to those of the bone [2]. It has been also consistently observed that the rate of tissue generation is proportional to the curvature (and the amount) of the surface [3] on which it grows. Thus, TPMSS can effectively be used as a porous scaffold thanks to their high surface-to-volume ratio. Moreover, TPMSS mechanical and physical properties provide possibilities in a variety of engineering applications: From lightweight construction elements and battery electrodes to photonic crystals [4,5]. These diverse structural and functional applications give the motivation for an accurate characterization of such metamaterials.

One of the challenges in the usage of TPMSS is the choice of a suitable geometry for a specific application. Yang et al. [6] have reported that the variation of the TPMSS unit cell size and wall thickness (WT) provides an opportunity to tune their elastic modulus, strength, and energy absorption. Moreover, in contrast to the strut-based lattice structures, this can be achieved without loss of structural integrity.

Another challenge is the production of defect-free, accurate TMPSS with low deviation from the intended geometrical models. This is particularly challenging in the case of metal PBF (powder bed fusion) manufacturing. The internal defects (gas pores, lack-of-fusion defects) as well as dimensional inaccuracies and excessive surface roughness are characteristic for EBM and LPBF processes [7,8]. It should be mentioned that overall surface topography together with physicochemical properties determine the biocompatibility of the implants at early stages of implant integration. Though commonly presented in the literature, averaged roughness parameters do not adequately represent the variety of the surface topography features important at different stages of the implant integration [9,10,11,12,13,14]. The detailed studies of the implant surface topography are commonly carried out using, for example, tactile profilometry, optical methods, or atomic force microscopy. However, the majority of the topography assessment methods are not suitable for lattices and other lightweight structures having the elements obscured from direct observation.

Finally, the inner structure of the TPMSS consists of curved channels. Therefore, the cleaning process of such structures from residual powder is also a highly challenging task. For example, removing residual powder from porous EBM parts is performed by a special powder recovery system (PRS) using precursor powder suspended in the jet of the compressed air. However, the efficiency of this method, namely the depth of powder blast penetration, is largely affected by the channel aspect ratio defined by the structure geometry, as well as by the size and type of the unit cells [15]. Other ways to treat the whole volume of the porous specimen are chemical and electrochemical etching (CE, ECE). These methods have been widely investigated by some researchers in order to improve some morphological characteristics (in particular surface roughness) of AM-samples [16,17]. However, such etching is often anisotropic and can lead to wall degradation, especially at the periphery of the samples. This can significantly affect the mechanical performance of the part.

X-ray Computed tomography (XCT) has already proved to be one of the best tools for non-destructive analysis of lattice and cellular structures [8,18,19]. It has been reported that XCT can detect the structural defects originated from the manufacturing process, as well as analyze the deflection of the geometry of manufactured components against their computer aided design (CAD) models [4]. Such knowledge can be further used to optimize the manufacturing process, reducing the defects, and leading to better geometry fidelity. Most importantly, XCT allows not only imaging but also extracting quantitative information about geometrical accuracy [20], manufacturing defects [21], surface texture [22,23,24], interconnectivity of the pores (see [25]), etc. However, there are few challenges in the analysis of the CT data of TPMSS. As mentioned above, the morphology of the structure even after the cleaning procedure can lead to retained powder (RP). The segmentation of the powder-containing regions is impossible with standard tools due to several reasons: (1) The identical gray level of the powder particles and wall regions limits the application of the global thresholding [26]; (2) the morphological filters or Watershed segmentation tools could modify the surface profile as well as the thickness of the walls, bringing additional error to the analysis.

Another challenge is the quantitative assessment of material loss caused by CE. The presence of RP inside TPMSS can hinder the uniform penetration of etchant, resulting in the gradient of the wall thickness (WT). Inhomogeneous CE can potentially cause anisotropy of the mechanical properties and unpredictability of the behavior under load condition. In this case, the mean WT value or the overall WT distribution determined by standard procedures are not informative and more sophisticated analysis is required.

In this work, we focus on XCT characterization of sheet-based TPMSS manufactured using EBM from Ti-6Al-4V. We present novel strategies and workflows for quantitative XCT image analysis of metallic TPMSS and functionally graded porous structures (FGPS). The procedures presented in this work allow obtaining information about the production and post-processing of AM structures. This information can be integrated into the optimization of the manufacturing process, and into the numerical simulations of the properties for the resulting structures [27]. The detailed investigation of the correlation between morphological characteristics, materials properties, and structure performance is not in the scope of this work and will be discussed in the follow-up publications.

## 2. Materials and Methods

### 2.1. Samples Design and Production

Two types of gyroid TPMSS (with constant and with functionally graded porosity) [4] were investigated. The gyroid surface was generated according to the following equation:

sin(kx)cos(ky) + sin(ky)cos(kz) + sin(kz)cos(kx) = 0(1)

where parameter k controls the unit cell size. In this study, k = 2 was chosen for TPMSS specimens with constant porosity. To make the gradient structure, k was defined as a function of one Cartesian coordinate, in our case z:

k(z) = 1 + jz(2)

**Figure 1:**
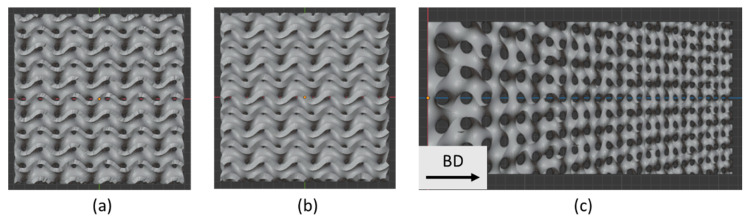
Models of the gyroid TPMSS with nominal WT of 0.4 mm (**a**), 0.6 mm (**b**), and gyroid-based functionally graded porous scaffold (FGPS) (**c**). BD indicates the build direction during manufacturing.

The scaling factor j was selected to be 0.05. The models of the designed structures are shown in Figure 1.

**Figure 2:**
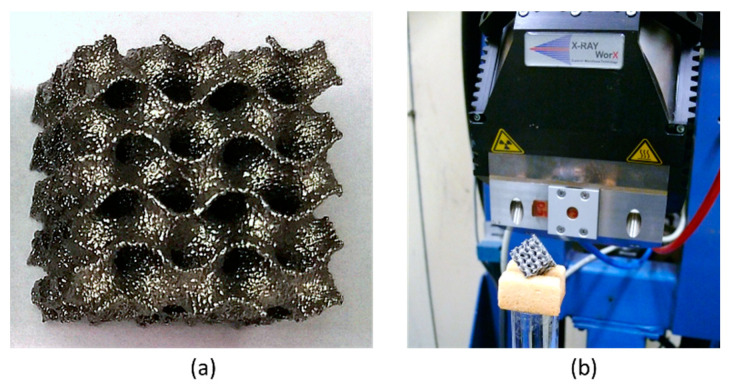
Photographs of (**a**) specimen with nominal WT of 0.4 mm; (**b**) the XCT experimental setup.

All specimens were manufactured from a Ti-6Al-4V alloy using an ARCAM A2 EBM machine by ARCAM, EBM (Mölnlycke, Sweden). The precursor powder was purchased from ARCAM EBM. The layer thickness was set to 50 µm and the process temperature was set to 720 °C. Standard process parameters for Ti-6Al-V from the ARCAM system library were used (the “Melt Theme” of the ARCAM EBM process library). The manufactured specimens (Figure 2a) were separated from the start plate and cleaned from the residual semi-sintered powder using a standard ARCAM Powder Recovery System.

Cubic specimens with constant porosity were designed with two different WT of 0.4 mm and 0.6 mm resulting in the specimens (15 mm × 15 mm × 15 mm) with relative material apparent density of 50% and 42%, respectively. In the following text, we name these specimens as 2 × 04 and 2 × 06, according to the parameter k and their nominal WT.

The specimen with gradient porosity was manufactured using a model with zero thickness and hence its WT was purely controlled by the electron beam application strategy (current, surface speed). The standard “Wafer Theme” from the ARCAM EBM process library was used, leading to the smallest WT achievable with EBM.

The removal of the precursor powder retained inside the structure after manufacturing is a critical step in the manufacturing chain. All specimens were subjected to a standard PRS cleaning for 10 min. Additionally, one specimen with constant porosity for each WT was chemically etched (CE) according to the following procedure: A 2 × 04 specimen was immersed in a 1% HF:10% HNO_3_ water solution 14 times for 3 min each, without solution renewal. The 2 × 06 specimen was immersed into the solution of the same composition 12 times for 3 min. It is known that the intensity of the etching decreases with time due to the formation of stable and soluble titanium hexafluoro complexes [27]. Therefore, in the case of the 2 × 06 specimen, the chemical solution was renewed after 12 and 24 min of etching. After each immersion step, the specimens were rinsed first with demineralized water and then with ethanol, and finally, air-dried.

### 2.2. X-ray Computed Tomography

The X-ray computed tomography (XCT) measurements were performed using a microfocus X-ray tube XWT-225-SE (maximum voltage 225 kV, (X-RAY WorX, Garbsen, Germany) from X-Ray WorX GmbH and an XRD1620 detector (CsI scintillator, 2048 × 2048 pixel, from PerkinElmer Inc., Waltham, MA, USA) with in-house built case and cooling system (Figure 2b). The tube voltage and a tube current were set to 120 kV and 120 μA respectively. The effective voxel size was (15.3 µm)^3^. The reconstruction of 3D volumes from 2D projections was carried out by in-house software using a filtered back-projection algorithm. Specimens were scanned with the cube diagonal parallel to the rotation axis (see Figure 2b), in order to reduce the influence of cone-beam artifacts.

## 3. Results and Discussion

### 3.1. Virtual Powder Removal

**Figure 3:**
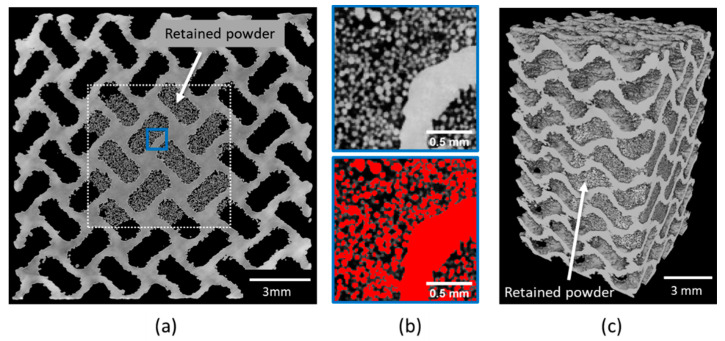
(**a**) Reconstructed XCT slice of the 2 × 04 specimen subjected to PRS showing the RP particles; (**b**) Magnified region indicated by blue rectangle in (**a**) and the result of the automatic thresholding using a standard Otsu’s method; (**c**) 3D rendering of the reconstructed TPMSS with the RP inside.

Retained powder (RP) is one of the limitations for the application of cellular structures with small pore size and is especially critical in medical implants [16]. The efficiency of the loose powder removal techniques can be non-destructively investigated by XCT. An example of XCT reconstructed slice of 2 × 04 specimen (Figure 3a) shows RP particles inside the structure. Obviously, the amount of RP depends on the pore and structure sizes. As it can be expected, the efficiency of the narrow channel cleaning by PRS drops with the distance from the specimen’s outer surface, leaving the powder residue mainly in the center of the specimen [15]. As stated above, it is challenging (and sometimes impossible) to remove all RP by conventional methods. The challenge is so formidable, that so far no perfect solution is available. However, vibration-assisted methods indicate that although damage can occur in beam-based lattice structures, such methods can be well suited for the TPMSS [7].

The RP prevents precise analysis of the XCT data, as such data should be segmented prior to further evaluation. The apparent density of the powder is smaller than the density of the bulk material (due to inter-particle void volume and internal porosity). Furthermore, if scanned with the low resolution, it could have had a sufficient contrast to be segmented by automatic thresholding [28]. In our case, the segmentation of the powder-containing regions is a challenging task. The resolution achieved allows distinguishing the individual powder particles that have the same gray value distribution in the reconstructed slices as the scaffold (Figure 3b). This limits the application of standard tools such as global thresholding [26]. One of the solutions could be the downscaling of the data, which would simulate the CT scan with lower resolution. However, this would cause loss of information about the surface topography as well as lead to additional error in the WT calculation. Therefore, we used a deep learning (DL) algorithm to solve this segmentation problem.

For the segmentation of the scaffold, we employed a 2D fully convolutional neural network (CNN) [29] implemented as a plugin in Fiji ImageJ (Version 1.53c) [30]. To perform segmentation, the CNN must undergo an iterative training procedure. Eight slices (with and without RP) were used for the training session. On these slices, the areas with RP were manually segmented and the rest of the scaffold was segmented using an advanced surface determination algorithm implemented in VG Studio Max (Version 3.4.3, Volume Graphics GmbH, Heidelberg, Germany) [31]. The CNN was trained with 500 iterations. The number of iterations usually depends on the diversity of the datasets. By tracking the convergence of the validation and training errors, we found that 500 iterations were optimal for the investigated datasets.

**Figure 4:**
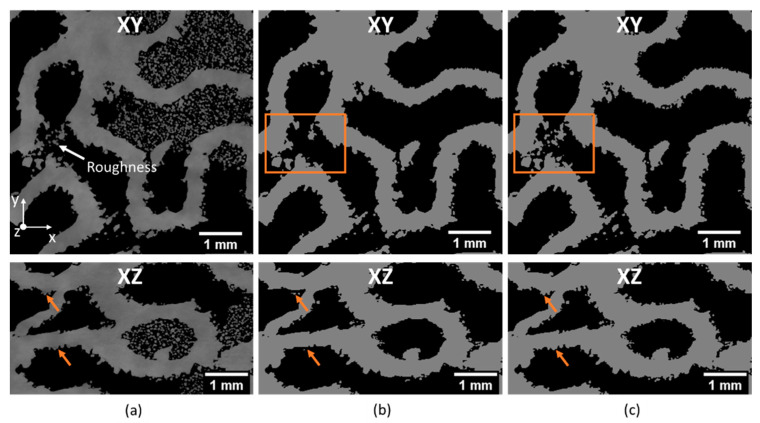
(**a**) A 2D reconstructed XY and XZ slices; (**b**) the result of the 2D DL segmentation of the scaffold only on XY planes; (**c**) the result of the combined segmentation on XY, XZ, and YZ planes. Orange boxes and arrows indicate the critical regions with surface roughness being wrongly segmented in (**b**).

**Figure 5:**
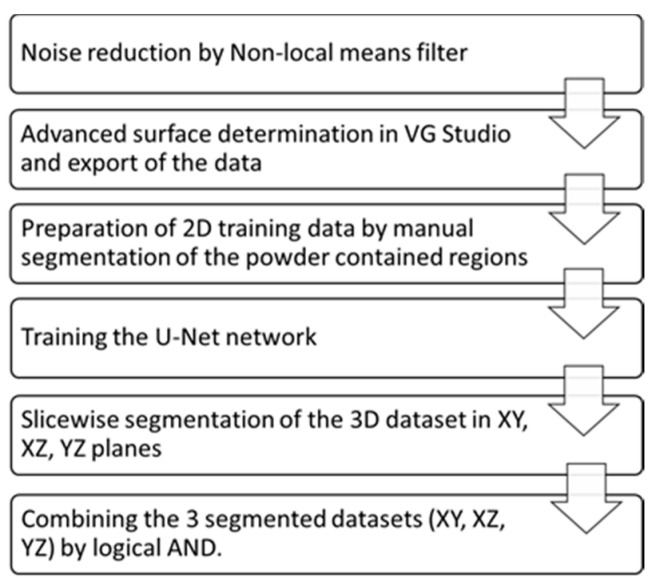
The description of the workflow used for segmentation of the RP.

The application of the 2D network only on XY slices resulted in under-segmentation of the scaffolds surface. In this case, the surface roughness of walls parallel to the XY plane (indicated by arrows on XZ slices, Figure 4a) appears as loose powder on the XY planes and is classified by the CNN as powder (Figure 4b). To overcome this issue, the segmentation procedure of every dataset was carried out in three planes (XY, XZ and YZ), followed by the logical operation AND on the three segmented volumes (Figure 4c). As a result, the scaffold was segmented with overall accuracy exceeding 95%. The accuracy was evaluated by pixelwise comparison of the ground truth (manually segmented) with the result of DL segmentation. The workflow is presented in Figure 5.

It is highly important to note that the presented methodology of using a 2D CNN instead of 3D CNN allowed achieving a high segmentation accuracy at a low cost. Typically, the amount of required training data is significantly smaller for the 2D networks and their computational efficiency is much higher.

### 3.2. Effect of Wall Orientation

The thickness of a powder-based AM component can vary depending on its angle to the build direction. Therefore, a calculation of an average WT within the specimen would imply a loss of information. A different WT of horizontal (within the deposited layers) and vertical (along the build direction) elements in TPMSS can lead to unwanted (and anyway uncontrolled) anisotropic mechanical properties of the component.

**Figure 6:**
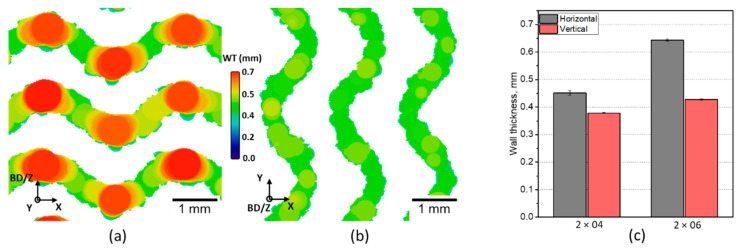
Comparison of the WT in the horizontal (printed normal to BD) and vertical (printed parallel to BD) sections: (**a**) Example of the CT-slice containing the horizontal wall of the 2 × 06 specimen; (**b**) example of the CT-slice containing the vertical wall of the 2 × 06 specimen; (**c**) quantitative comparison of WT in 2 × 04 and 2 × 06 specimens. BD indicates the build direction.

XCT allows investigating the distribution of the WT for the elements printed at different angles to the build direction. In this case, the WT was quantitatively analyzed for all near-horizontal and near-vertical walls in both the 2 × 04 and 2 × 06 specimens (Figure 6) using the algorithm of volumetric local thickness [32] implemented in the BoneJ plugin [33] for Fiji ImageJ (Version 1.53c). The algorithm is based on fitting maximal spheres to every point of the structure. It is important to note that the used algorithm of local thickness evaluates the surface roughness of a wall as an intersection of objects of different size (the wall itself and the attached powder particles). The local thickness of such a joint structure is determined by the diameter of the largest sphere fitted to the wall region and smaller spheres fitted to the roughness regions. Consequently, the presence of partially molten powder particles attached to the surface can lead to an underestimation of the mean WT. Therefore, only local thickness values higher than 150 µm were included in the analysis. We experimentally found the value of 150 µm to be the optimal to exclude the contribution of those powder particles attached to the surface.

A significant difference in the thickness of horizontal and vertical walls was observed for both specimens (Figure 6c). In the 2 × 06 specimen, the vertical elements appear up to 180 µm (around 30%) thinner than the nominal WT while horizontal elements almost coincide in thickness with the nominal value.

The effect of the wall orientation (with respect to the build direction) on the WT and roughness in the PBF-AM has a known origin but is hard to control. The thickness of the printed walls not only depends on the CAD model, but also on the size of the melt pool and on the direction of heat dissipation. The “Melt Theme” in ARCAM EBM used in this work optimizes the parameters of the electron beam for bulky specimens i.e., considering the large cross-sections of the printed part withing the build plane. The cross-section size of TPMSS vertical and horizontal walls (within the build plane) differs significantly, leading to different temperature gradients and hence different sizes of the melt pool. In the vertical walls, the melt pool is supported by the solid material in the layers below, leading to a fast heat dissipation and swells mainly in the horizontal plane, capturing some powder particles. This phenomenon leads to some waviness of the vertical wall sides (with characteristic “wavelengths” proportional to the used layer thickness) and irregular roughness caused by the partially fused powder particles. In the horizontal walls, the metal solidifies over underlying powder layers. While in EBM, such powder layers are pre-sintered, rather than loose as in LPBF, liquid metal from the melt pool is able to “leak” through the powder bed. This phenomenon results in excessive surface roughness and even stalactite-like structures.

It is important to consider the angular dependence of WT since it can cause anisotropic mechanical properties and unpredictable mechanical performance of TPMSS. One approach to reduce the influence of the temperature gradients and hence the difference in the WT is to build the cubic TPMSS tilted by 45° (i.e., to align the main cube diagonal with the build direction). Another approach is to directly compensate for the expected WT deviation in CAD models. This, however, requires detailed research to define the exact correlation between build angles of the walls and their thickness deviations.

### 3.3. Wall Degradation after Etching

**Figure 7:**
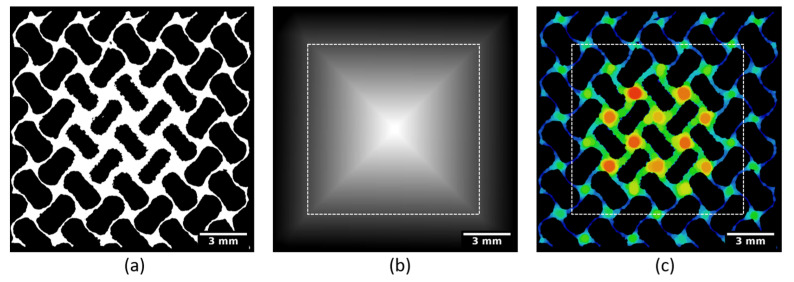
Schematic workflow generated for WT analysis of the gradient structures. (**a**) A binarized 2D slice of the 2 × 06 specimen after CE; (**b**) the Euclidean distance map; (**c**) the result of the WT analysis. The white square indicates one set of voxels having the same distance from the closest edge.

Post-manufacture chemical etching (CE) is often used on PBF lattice structures to reduce the surface roughness [34], which usually results in improvement of the mechanical properties [35]. In the case of sheet-based TPMSS, in addition to surface modification, CE can also be applied as a cleaning procedure dissolving the RP. However, the penetration of the fresh etchant into the deep voids is restricted and the solution etches the specimen periphery faster than the interior. This is visible in Figure 7a,c. This preferential etching leads to a gradient of the WT towards the center of the specimen and can cause the unpredictability of the mechanical performance.

**Figure 8:**
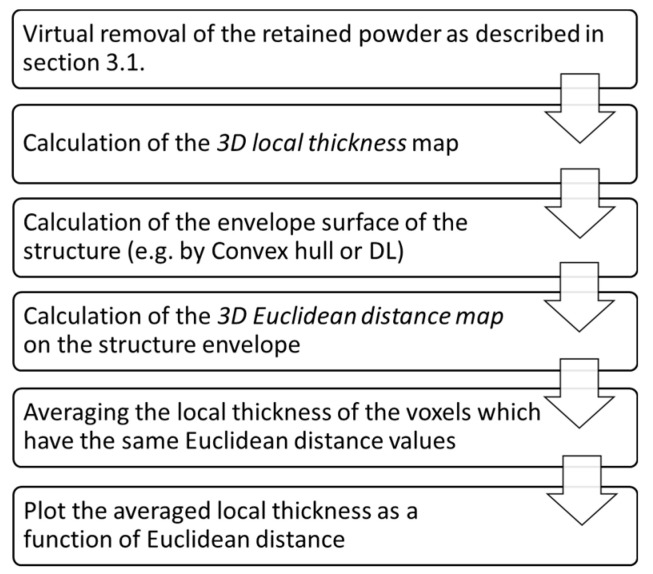
The description of the workflow used for the wall degradation analysis.

There is no ready solution for a XCT-based quantitative evaluation of the WT gradient, although it is highly important for the quality assessment as well as for the prediction of the mechanical behavior. Therefore, in this section, we present a method we developed to evaluate the wall degradation after CE. Figure 7a shows a binarized reconstructed XCT slice of the 2 × 06 specimen after CE (the RP is not shown). The WT clearly increases from the specimen periphery to its center. The overall mean WT determined by standard procedures is no longer relevant in such a gradient structure. Therefore, an adapted XCT image analysis workflow was developed to quantitatively evaluate the loss of material as a function of distance from the specimen periphery. This procedure will help to tune the CE parameters as a function of the porosity of the specimen. The procedure consists of the following steps (see Figure 7 and Figure 8):

1. Prior to the WT analysis, the scaffold was segmented from the loose powder using the algorithm described in the Section 3.1.
2. The envelope structure defined by the outer region of the specimen was generated. For simple convex objects, this can be done by calculation of the convex hull, which is the smallest convex shape containing all points of an object. For more complex geometries, the envelope surface can be identified by sophisticated DL algorithms.
3. The 3D Euclidean distance map was calculated on the convex hull of the structure (Figure 7b). This allows assigning every point of the structure the value of the shortest distance between this point and the background (specimen’s edge).
4. Finally, the average value of the local thickness was calculated for every set of voxels equidistantly located from the specimen’s edges (with the same Euclidean distance value, as indicated by the white dashed line in Figure 7b,c). This combination of complementary information on the WT and the Euclidean distance allows a quantitative characterization of the WT gradient in a direction from the specimen edges to the center. Figure 8 summarizes the workflow.

**Figure 9:**
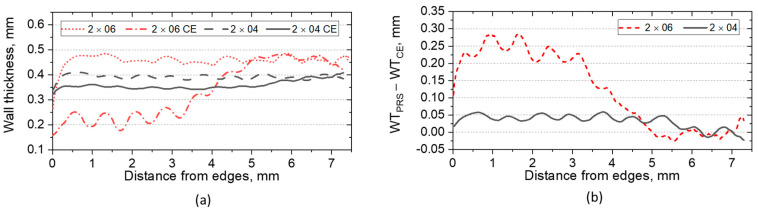
(**a**) WT as a function of distance to the edges; (**b**) the difference between as-build (PRS) and CE WT as a function of distance to the edges.

In both 2 × 04 and 2 × 06 specimens, the CE could not completely remove the RP from the structure. A small amount of powder remained in the center of specimens (see Figure 9) in the region where WT curves of as-build and CE specimens coincide: At a distance of around 5 mm for 2 × 06 and of around 6 mm for 2 × 04. The region where some powder is retained shows a linear increase of the WT to the region where the pores are completely filled with powder. A strong gradient of WT is created by the CE in the case of the 2 × 06 specimen, showing a reduction in the mean WT from around 470 µm (in as-build condition) to 200 µm (after CE). The large material loss (also seen in Figure 7a) is caused by the aggressive etching strategy, i.e., the renewal of the chemical solution leads to a more intensive etching but still does not completely remove the RP.

**Figure 10:**
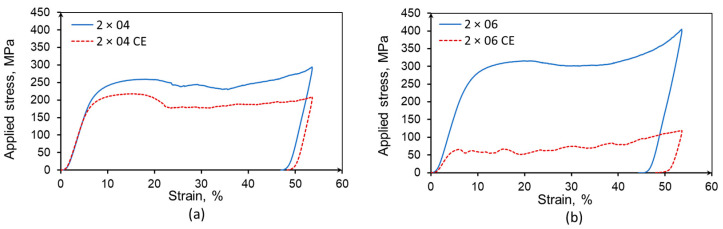
Stress–strain curves obtained during compression tests on 2 × 04 (**a**) and 2 × 06 (**b**) specimens in as-build condition and after CE.

The effect of the wall degradation on the mechanical performance of the specimens was investigated by compression tests (Figure 10) conducted at 20 °C according to ISO 13314:2011 [36] and using a strain-rate of around 5.5 × 10^−4^. Both specimens show a reduction of the yield stress as well as of the plateau-stress (defined between 20% and 40% strain).

### 3.4. Surface Roughness Analysis

**Figure 11:**
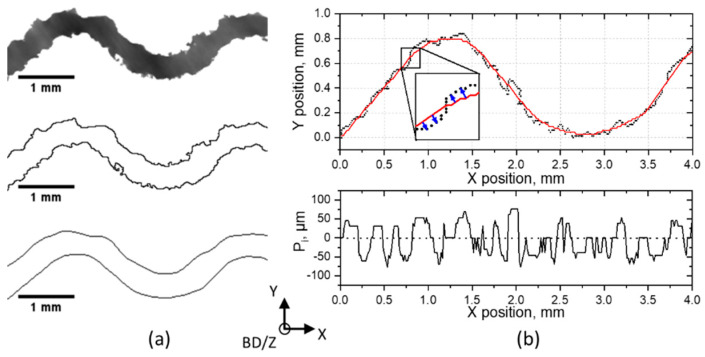
Evaluation of the surface roughness. (**a**) A 2D cross-section extracted from the vertical wall of 2 × 04 specimen (top), the profiles of both sides of the wall (middle), the profiles after low-pass filtering (bottom); (**b**) example showing the original (black points) and filtered profiles (continuous red line) and the resulting roughness profile (bottom). BD indicates the build direction (normal to the layer surfaces).

Surface roughness is another critical parameter for the mechanical performance of TPMSS, especially under cyclic load [37,38]. XCT must be used to determine surface roughness in lattice structures, as no other tactile or laser technique can interrogate their interior. However, the XCT determination of the surface roughness on the curved surface is a challenging task. Therefore, we present a multistep procedure we developed to determine roughness of the walls based on the XCT data. In order to exclude the influence of building orientation and compare different specimens, only vertical walls (parallel to BD) in regions not containing the RP were analyzed. First, 2D cross-sections of the vertical walls were extracted for every specimen (Figure 11a). Then the advanced surface determination tool available in VG Studio Max [31] was employed to identify the surface of the wall. The segmented walls were then exported, and an in-house-developed Fiji ImageJ routine was used for further analysis. Wall profiles were created on both sides of the segmented walls (Figure 11a middle). To identify the base line, the profiles were processed with a low-pass filter (Figure 11a bottom represents the filtered profiles).

To obtain the roughness profile, a simple subtraction of the original and filtered profiles would not be accurate in the regions with non-zero slope. Instead, the distance between the baseline and the profile at each point was calculated along the normal to the filtered profile. This procedure is shown in the inset in Figure 11b top, where the red continuous curve represents the filtered profile, and the points are the (discrete) measured surface values. Re-entrant features were not considered in the analysis. The resulting roughness profile is shown at the bottom of Figure 11b.

The average roughness parameter P_a_ was calculated according to Equation (3), and the resulting values are presented for all specimens in Table 1.

(3) $$P_{a}=\frac{1}{n}\overset{n}{\underset{i=1}{\sum }}\left|P_{i}\left(x\right)\right|$$

In order to get a statistically correct result and estimate a statistical error, the P_a_ value was calculated for ten 2D wall cross-sections, resulting in 20 roughness profiles for every specimen. The error is represented by the standard deviation of 20 measurements.

It is shown in literature that the arithmetic roughness (R_a_) for EBM-manufactured vertical walls (or struts) ranges from 20 µm to 50 µm [39,40]. In our case, similar values (slightly below 40 µm) were obtained for the as-build 2 × 04 and 2 × 06 specimens. A slight reduction of the roughness was observed for the 2 × 04 CE specimen. In the case of the 2 × 06 CE specimen, the etching procedure could significantly reduce the roughness. However, as it was shown in the previous section, the periphery wall degradation, and at the same time the poor cleaning results, indicate the necessity of an improvement of the CE process. The combination of XCT data with advanced algorithms for surface topography determination provides a valuable method for the study (in 3D) and the improvement of the biocompatibility of lattice structures.

### 3.5. Analysis of the FGPS Lattice Structure

**Figure 12:**
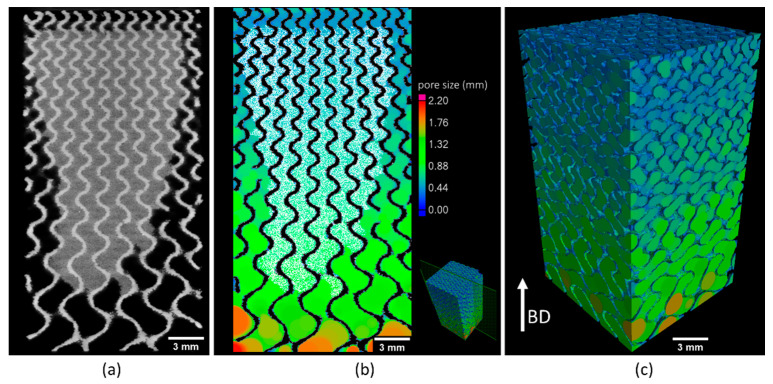
(**a**) 2D slice of a 3D reconstruction of a FGPS structure; (**b**) 2D slice showing the result of 3D pore size analysis (the white area represents the entrapped powder particles); (**c**) 3D rendering of the pore size distribution along the build direction (BD).

As mentioned above, one of the approaches to mimic the bone structure is the manufacture of functionally graded porous structures along a particular direction. In this case, the problem of RP is also present and needs to be considered when optimizing the size of the smallest pore (see Figure 12). A FGPS specimen with a porosity gradient in the build direction was investigated, combining the approaches described above. The pore size distribution was analyzed in the direction of the gradient.

**Figure 13:**
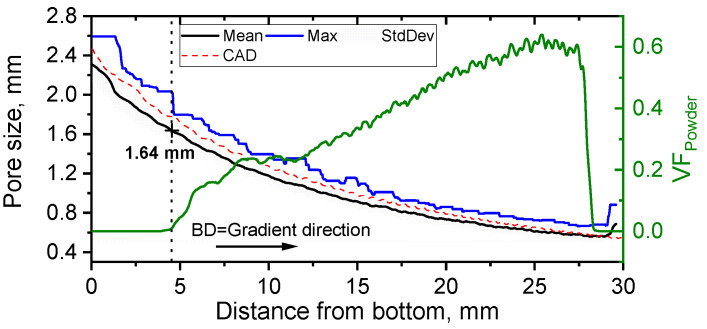
Pores size and volume fraction of entrapped powder (VF_powder_) as a function of FGPS height.

The volume fraction of RP was calculated as a ratio of the volume occupied by powder to the volume of the pores. The resulting mean and maximum pore size distribution as well as the volume fraction of RP are shown in Figure 13. It can be seen that the efficiency of the powder removal process is only satisfactory for pore sizes up to 1.6 mm. Lower porosity leads to the presence of RP, and, consequently, requires advanced cleaning procedures.

The theoretical pore size distribution was also analyzed using a model of the FGPS with a nominal WT of 0.25 mm. The theoretical distribution is in a good agreement (within a standard deviation) with the experimental one. Interestingly, no fluctuations of the pore size curve were observed, indicating no thickness difference between horizontal and vertical walls.

To conclude, we can state that although more detailed studies are necessary to elucidate this aspect further, certain general criteria can be formulated for the design of FGPS manufactured by PBF AM: The smallest feasible cell size is determined not only by the manufacturing process and by the spatial resolution achievable with a particular machine, but also by the possibility to effectively remove the RP. In addition, powder cleaning efficiency depends on the overall dimensions of the porous sections and design of the implant. For example, the efficiency of the PRS system standard for EBM strongly falls as a function of depth in any structure: It is hard for the airflow to penetrate deep into the long channels filled with RP or solid sections of the component [15]. Multiple additional technologies supporting easier powder recovery should be applied with care, as some of them can negatively impact the mechanical properties of the structures (e.g., [7]). Until more reliable industry-accepted powder recovery methods are in place, the most reliable way to ensure thorough cleaning is to manufacture sets of structures with fixed TPMS cell properties and to use standard methods, such as EBM-PRS. Routines developed in this work for non-destructive characterization of the FGPS structures are an effective way to assess the quality of manufacturing and post-manufacturing processes, being a prerequisite for further technology improvement.

## 4. Conclusions

X-ray computed tomography (XCT) is the only tool for non-destructive and quantitative morphological characterization of additively manufactured triply periodic minimal surface structures (TPMSS). We showed that the challenge of segmenting the retained powder can be efficiently tackled by employing deep learning algorithms. The presented methodology of using a 2D convolutional neural network (CNN) instead of a 3D CNN allowed achieving high segmentation accuracy at a low cost (small amount of training data and high computational efficiency). This step allowed further metrological analysis of the TPMSS:

(1) The wall thickness showed a clear dependence on the wall orientation (with respect to the build direction): The horizontal walls (parallel to the build plane) appear up to 30% thicker than the vertical ones. Quantification of this known phenomenon is extremely important since it can cause a strong anisotropy of the mechanical properties.
(2) The chemical etching (CE) as a postprocessing technique was characterized assessing its efficiency in removing the retained powder, its effect on the wall thickness, and the surface roughness. The wall thickness gradient (from the edges to the specimen’s center) was quantified using a developed methodology by combining the local thickness algorithm with the Euclidean distance transform. Strong gradient and large material loss were found in the specimen subjected to aggressive etching. In both specimen types, the powder could not be fully removed. This indicates that CE cannot be used as a standalone cleaning tool. Moreover, the material loss resulted in a reduction of the mechanical properties under compressive load. We found a positive effect of the CE on surface roughness. Our surface roughness analysis method allowed investigating curved walls of the TPMSS and could disclose a decrease of the average roughness after the CE.
(3) Finally, the pore size distribution in functionally graded TPMSS was analyzed and correlated to the amount of retained powder. This procedure can be used to evaluate the cleaning routines and determine the minimum pore size compatible with a powder-free part, which is the premise for the optimization of the manufacturing process and for utilization of TPMSS in the biomedical sector.

## Figures and Tables

**Table 1:** Averaged roughness parameter P_a_ for as-build and CE specimens.

**Specimen**	2 × 04	2 × 04_CE	2 × 06	2 × 06_CE
**P_a_, µm**	36.6 ± 3.1	30.8 ± 3.5	38.3 ± 4.6	16.4 ± 2.7

## Data Availability

The data presented in this study are available on request from the corresponding author. The data are not publicly available as they also form part of an ongoing study.
